# Sulforaphane Inhibits Foam Cell Formation and Atherosclerosis via Mechanisms Involving the Modulation of Macrophage Cholesterol Transport and the Related Phenotype

**DOI:** 10.3390/nu15092117

**Published:** 2023-04-28

**Authors:** Shiyan Liu, Yuan Zhang, Xiangyu Zheng, Ziling Wang, Pan Wang, Mengdi Zhang, Mengfan Shen, Yongping Bao, Dan Li

**Affiliations:** 1 Department of Nutrition, School of Public Health, Sun Yat-sen University, Guangzhou 510080, China; 2 Guangdong Provincial Key Laboratory of Food, Nutrition and Health, Guangzhou 510080, China; 3 Guangdong Engineering Technology Center of Nutrition Transformation, Guangzhou 510080, China; 4 Department of Geriatrics, The Third Affiliated Hospital of Guangzhou Medical University, Guangzhou 510150, China; 5 Norwich Medical School, University of East Anglia, Norwich NR4 7UQ, Norfolk, UK

**Keywords:** sulforaphane, isothiocyanate, atherosclerosis, cardiovascular disease, macrophage, cholesterol, foam cell, Nrf2, cruciferous vegetable, ApoE-deficient mice

## Abstract

Sulforaphane (SFN), an isothiocyanate, is one of the major dietary phytochemicals found in cruciferous vegetables. Many studies suggest that SFN can protect against cancer and cardiometabolic diseases. Despite the proposed systemic and local vascular protective mechanisms, SFN’s potential to inhibit atherogenesis by targeting macrophages remains unknown. In this study, in high fat diet fed ApoE-deficient (ApoE^−/−^) mice, oral SFN treatment improved dyslipidemia and inhibited atherosclerotic plaque formation and the unstable phenotype, as demonstrated by reductions in the lesion areas in both the aortic sinus and whole aorta, percentages of necrotic cores, vascular macrophage infiltration and reactive oxygen species (ROS) generation. In THP-1-derived macrophages, preadministration SFN alleviated oxidized low-density lipoprotein (ox-LDL)-induced lipid accumulation, oxidative stress and mitochondrial injury. Moreover, a functional study revealed that peritoneal macrophages isolated from SFN-treated mice exhibited attenuated cholesterol influx and enhanced apolipoprotein A-I (apoA-I)- and high-density lipoprotein (HDL)-mediated cholesterol efflux. Mechanistic analysis revealed that SFN supplementation induced both intralesional and intraperitoneal macrophage phenotypic switching toward high expression of nuclear factor erythroid 2-related factor 2 (Nrf2), heme oxygenase-1 (HO-1) and ATP-binding cassette subfamily A/G member 1 (ABCA1/G1) and low expression of peroxisome proliferator-activated receptor γ (PPARγ) and cluster of differentiation 36 (CD36), which was further validated by the aortic protein expression. These results suggest that the regulation of macrophages’ cholesterol transport and accumulation may be mainly responsible for SFN’s potential atheroprotective properties, and the regulatory mechanisms might involve upregulating ABCA1/G1 and downregulating CD36 via the modulation of PPARγ and Nrf2.

## 1. Introduction

Atherosclerosis constitutes the main underlying pathology of cardiovascular diseases (CVDs). In all the stages of atherosclerosis, macrophages play an important role. An elevated level of circulating low-density lipoprotein-C (LDL-C) is an established major risk factor for CVDs. It is gradually deposited in the subendothelial matrix and undergoes modifications such as oxidation and acetylation, which facilitate its subsequent uptake by macrophages, resulting in the formation of cholesterol-engaged ‘foam cells’, the hallmark of atherosclerosis [1,2,3]. Under chronic insult, the reparative and homeostatic functions of the plastic macrophages can be subverted, and cholesterol load may induce their functional shift, particularly to proatherogenic processes [3,4]. In these processes, redox stress and mitochondrial disturbances are elicited. They interact and contribute to macrophages’ polarization towards a proinflammatory phenotype and apoptosis, favoring the initiation and development of atherosclerosis. Therefore, the ability of phytochemicals and pharmaceutical agents to repress foam cell formation, as well as their antioxidant and mitoprotective properties, endows them with preventive and therapeutic potential regarding atherosclerosis [5,6,7,8,9].

The formation of foam cells is determined by lipid uptake, cholesterol esterification and cholesterol efflux, and in a state of atherosclerosis, the first two processes are increased, while the last is insufficient [8]. Lipid uptake by macrophages is primarily mediated by a scavenger receptor (SR)-mediated pathway. Although several SRs can bind to and internalize modified low-density lipoprotein (LDL), class A scavenger receptors (SR-A) and cluster of differentiation 36 (CD36) have been confirmed to account for approximately 75~90% of oxidized low-density lipoprotein (ox-LDL) internalization by macrophages [1,8], and the two SRs probably act in a complementary fashion [10]. The specific oxidized lipids present in internalized ox-LDL particles function as ligands for nuclear hormone receptor peroxisome proliferator-activated receptor γ (PPARγ) and upregulate the expression of CD36, and the positive feedback loop between them leads to a vicious cycle [11,12]. CD36 has been extensively demonstrated to possess the atherogenic property [12,13,14,15,16]. Additionally, a soluble form of CD36 (sCD36) in plasma has been demonstrated to represent a potential surrogate marker of atherosclerosis [12,17].

Decompensated cholesterol metabolism in macrophages in response to excessive cholesterol uptake may result in pathological processes [1]. Several macrophage transporters promote the efflux of lipids, such as ATP-binding cassette transporter A family member 1 (ABCA1), ATP-binding cassette transporter G1 (ABCG1) and scavenger receptor class B type 1 (SR-B1). ABCA1 mainly promotes the migration of intracellular free cholesterol into lipid-poor apolipoprotein A-I (apoA-I) particles, while ABCG1 tends to promote the migration of intracellular cholesterol and cholesterol esters into high-density lipoprotein (HDL) particles. Under lipid loading, ABCA1 is responsible for 40~50% of cholesterol efflux and ABCG1 is responsible for ~20%. Peripheral cholesterol can be transferred to the liver, where it can be converted into bile acid and excreted via the feces. This process is called ‘reverse cholesterol transport’ (RCT), and cholesterol efflux is the first step. Defective cholesterol efflux leads to the development of atherosclerosis [18,19]. Interventional and genetic studies, as well as research using gene manipulation techniques, have determined the inhibitory effect of ABCA1/G1-mediated macrophages’ cholesterol efflux on foam cell formation and atherosclerosis [20,21,22,23,24]. Additionally, several large studies have verified that there is an inverse correlation between the capacity of plasma HDL to mediate macrophages’ cholesterol outflow and arteriosclerotic cardiovascular disease (ASCVD) [25,26,27].

Epidemiological studies have indicated that a high intake of cruciferous vegetables correlates with a decreased risk of chronic diseases, and the beneficial effects have been largely attributed to high levels of glucoraphanin, a precursor for the isothiocyanate (ITC) sulforaphane (SFN). Preclinical studies and clinical trials have demonstrated that SFN exerts cardioprotective effects via antioxidant, anti-inflammatory and epigenetic mechanisms and regulates glucolipid metabolism [28]. Additionally, several important steps in the initiation and progression of atherosclerosis have been shown to be regulated by SFN, including endothelial dysfunction [29,30], monocyte adhesion to endothelial cells [31], vascular smooth muscle cells proliferation and platelet aggregation [32,33,34,35,36]. However, the impact of SFN on foam cell formation, the core event of atherosclerosis, has not been reported to date. Moreover, there is no study reporting the role of SFN in atherosclerotic plaque formation, despite the finding that it protects against neointima formation and vascular injury [29,30,31,32,33,34,35].

Dyshomeostasis is the hallmark of many metabolic diseases. It is usually manifested by oxidative stress, metabolic inflexibility and uncontrolled inflammatory reaction. The multifactorial feature of the diseases could be targeted with one single hit on the pleiotropic nuclear factor erythroid 2-related factor 2 (Nrf2) [37]. Meanwhile, simple lifestyle changes including elevated consumption of dietary Nrf2 activators are considered to have a profound impact on atherosclerosis. Nrf2 is closely linked to atherosclerosis. During its progression, Nrf2 signaling regulates multiple processes, such as cellular lipid and redox homeostasis, the formation of foam cells, macrophage phenotypic shifts and inflammation [38]. Intriguingly, Nrf2 displays both atheroprotective and atherogenic effects in rodent and rabbit models [38,39,40,41,42,43,44] and can both promote and inhibit foam cell formation via transactivating CD36 and inducing ABCA1/G1 expression, respectively [40,45,46,47,48,49,50]. Inconsistent findings for Nrf2’s role in CD36 expression and the negative regulation of SR-A by Nrf2 have also been reported [38,39,43,51]. SFN is the most potent naturally occurring inducer of Nrf2 and the acknowledged gold standard compound for other covalent Nrf2 activators [52]. Therefore, we are very curious about the role of SFN in cholesterol trafficking, the expression of related executors in macrophages and the consequent lipid accumulation and atherosclerosis.

PPARγ is one of the three peroxisome proliferator-activated receptor (PPAR) subtypes belonging to the nuclear receptor superfamily. It plays crucial roles in glucose and lipid metabolism, including the promotion of cholesterol efflux, mediating the control of cardiac function and modulating risk factors for CVD, and has therapeutic potential for metabolic diseases [53,54]. However, there is considerable evidence showing unfavorable effects of PPARγ on foam cell formation and atherosclerosis via the transcriptional activation of CD36 and its positive correlation with ectopic lipid accumulation, including that observed in atherosclerosis [8,55,56]. Moreover, as a PPARγ agonist and insulin sensitizer, the antidiabetic drug thiazolidinedione (TZD) has also been reported to increase the risk of cardiovascular events and lead to fat redistribution to peripheral sites [56,57,58,59]. More confusingly, crosstalk and even positive feedback between PPARγ and Nrf2 have been proved to exist [57,60], but SFN-induced PPARγ downregulation has also been found in adipose and liver tissues/cells and suggested to account for SFN’s antiobesogenic, antiadipogenic and antilipogenic activities [61,62,63,64].

The purpose of this study is to investigate whether SFN treatment could influence macrophage foam cell formation and atherosclerosis and explore the potential mechanistic contribution of CD36, ABCA1/G1 expression and the involvement of PPARγ and Nrf2.

## 2. Materials and Methods

### 2.1. Antibodies and Reagents

R,S-sulforaphane was purchased from LKT Laboratories (S8044, Saint Paul, MN, USA). Water-soluble tetrazolium salt-1 (WST-1) Cell Proliferation Reagent was obtained from Roche (Shanghai, China). Ox-LDL was purchased from Yiyuan (YB-002, Guangzhou, China). Oil-Red-O and 2′,7′-dichlorodihydrofluorescein diacetate (DCFH-DA) were purchased from Sigma (D6883, Saint Louis, CA, USA). Dihydroethidium (DHE) was purchased from Molecular Probes (Eugene OR, USA). 4′,6-diamidino-2-phenylindole (DAPI) was purchased from Beyotime Biotechnology (Shanghai, China). 5,5′,6,6′-tetrachloro-1,1′,3,3′-tetraethylbenzimi-dazolylcarbocyanine iodide (JC-1) was procured from Enzo Life Sciences, Inc. (ENZ-52304, New York, NY, USA). Goat anti-mouse IgG was purchased from Santa Cruz Biotechnology (Dallas, TX, USA). Goat antirabbit IgG H&L (HRP) was purchased from Abcam (Cambridge, UK). Alexa Fluor 647 antirat IgG, Alexa Fluor 546 antirabbit IgG and Alexa Fluor 594 anti-mouse IgG were purchased from Invitrogen (Waltham, WA, USA).

### 2.2. Animals and Treatments

All the animal experiments were approved by the Institutional Animal Ethics Committee of Sun Yat-sen University (No.2022-044). ApoE^−/−^ mice (males, 8 weeks old) with a C57BL/6J background were obtained from the Vital River Laboratory Animal Technology Corporation Co., Ltd., Beijing. All the mice were housed in a pathogen-free animal facility under controlled temperature (23 ± 2 °C) and a 12 h light/dark cycle with free access to water and a high-fat diet (21% milk fat, 0.15% cholesterol, MD12015, Medicines Ltd., China) during the treatment. Thirty-six ApoE^−/−^ mice were randomly assigned into three groups (*n* = 12 per group): (1) mice treated with 0.5% carboxymethylcellulose sodium (the Ctrl group), (2) mice treated with SFN at 5 mg/kg bw (the SFN-L group) and (3) mice treated with SFN at 15 mg/kg bw (the SFN-H group). SFN was dissolved in 0.5% carboxymethylcellulose sodium, administered by oral gavage every other day for 16 weeks. The weight of the mice was recorded weekly, and their daily food intake was also calculated. At the end of the experiments, fasting blood samples were collected, and the serum was isolated after centrifugation at 1000× *g* for 10 min and then stored at −80 °C. Peritoneal macrophages (PMs) were isolated and seeded in petri dishes for subsequent experiments. The whole aorta was carefully isolated under the microscope. The hearts were embedded in optimum cutting temperature (OCT) compound. Aortic sinus sections were collected from the initiation to the disappearance of aortic valves. Three randomized aortic sinus sections from each mouse were then used for quantitative analysis.

### 2.3. Quantification of Atherosclerotic Lesions

Oil Red O staining and quantification were applied to analyze the lesion size for the whole aorta and aortic sinus. For aortic tree analysis, freshly isolated aortas were trimmed free of fat and cut longitudinally from the ascending arch to the iliac bifurcation before en face staining. For aortic sinus analysis, hearts with aortic roots were harvested from mice, embedded in OCT compound and sectioned to an 8 μm thickness. Serial sections throughout the aortic sinus were obtained from each mouse and stained with Oil Red O. The necrotic cores in lesion areas were determined by hematoxylin and eosin (H&E) staining with aortic root cross-sections. The images of the atherosclerotic lesions were captured with a microscope (DFC 425C, Leica, Wetzlar, Germany) and analyzed by using ImageJ.

### 2.4. Serum Lipid Profile Analysis

The levels of total cholesterol (TC), high-density lipoprotein cholesterol (HDL-C), LDL-C and triglycerides (TGs) in mouse serum were determined using quantification kits (Nanjing Jiancheng Bioengineering Institute, Nanjing, China) according to the manufacturer’s instructions.

### 2.5. Immunofluorescence

Aortic root cryosections were fixed in 4% paraformaldehyde and permeabilized in 0.05% Triton X-100. After blocking with 1% BSA at room temperature for 1 h, the sections were incubated overnight at 4 °C with primary antibodies: anti-CD36 (NB600-1423; 1:100; Novus, Minneapolis, MN, USA), anti-ABCA1 (NB400-105; 1:100; Novus), anti-ABCG1 (NB400-132; 1:100; Novus), anti-Nrf2 (GTX103322; 1:100; Gene Tex, Irvine, CA, USA), anti-PPARγ (sc-7273; 1:100; Santa Cruz Biotechnology) and anti-F4/80 (ab6640; 1:100; Abcam), followed by incubation with corresponding Alexa Fluor conjugated secondary antibodies for 1 h at room temperature. The nuclei were counterstained with DAPI. A negative control lacking primary antibodies was performed at the same time. Images were captured using a confocal laser scanning microscope (TCS SP5, Leica, Wetzlar, Germany).

### 2.6. Dihydroethidium (DHE) Staining

Vascular superoxide anion production was evaluated by the DHE staining of aortic root sections. Briefly, after incubation with 2% Triton X-100 for 10 min, slides were stained with 5 μM DHE in Krebs solution in the dark at 37 °C for 30 min and immediately evaluated by confocal fluorescence microscopy. The percentage of red fluorescent signal was analyzed using ImageJ.

### 2.7. Mouse Peritoneal Macrophage Isolation and Culture

After 3 days of the intraperitoneal injection of 1 mL of 4% thioglycolate solution, PMs were isolated from ApoE^−/−^ mice by peritoneal lavage with sterile PBS and seeded into plates. After incubation for 3 h to allow adherence, nonadherent cells were removed by washing with PBS, and the adhered macrophages were cultured in RPMI 1640 medium supplemented with 10% FBS.

### 2.8. THP-1 Cell Differentiation and Culture

The human monocyte leukemia cell line (THP-1) was obtained from the American Type Culture Collection (ATCC, Manassas, VA, USA). The cells were cultured in RPMI 1640 medium containing 10% fetal bovine serum (FBS, Gibco, Gaithersburg, MD, USA), 100 units/mL penicillin and 100 µg/mL streptomycin (Gibco, Gaithersburg, MD, USA) in humidified air containing 5% CO_2_ at 37 °C. In all the experiments, THP-1 monocytes were incubated with 100 ng/mL phorbol 12-myristate 13-acetate (PMA, Abcam, Cambridge, UK) for 48 h to differentiate them into adherent macrophages before further treatment.

### 2.9. Cell Viability Assay

Cell viability was determined using the WST-1 assay. Differentiated THP-1 cells were seeded into 96-well plates (100 μL/well, 3 × 10^5^/mL) and incubated with a series of concentrations of SFN (2.5~100 μM) for 24 h. WST-1 reagent (10 μL) was then added to each well. After incubation at 37 °C for an additional 4 h, the absorbance at 450 nm was measured by using a microplate spectrophotometer (Bio-Tek Instrument Inc., Winooski, VT, USA).

### 2.10. Foam Cell Formation Assay

Differentiated THP-1 cells were seeded into 6-well plates (2 mL/well, 5 × 10^5^/mL) and pretreated with SFN or the vehicle (<1‰ DMSO) for 24 h and incubated with 50 μg/mL ox-LDL for 6 h. Subsequently, the cells were fixed with 4% paraformaldehyde for 20 min, then washed with PBS and stained with Oil Red O for 30 min at 37 °C. The foam cell morphology was photographed under an optical microscope (Carl Zeiss AG, Aalen, Germany). The Oil Red O positive area was measured using ImageJ.

### 2.11. Dil-oxLDL Uptake Assay

1,1′-dioctadecyl-3,3,3′,3′-tetramethyl indocarbocyanine perchlorate (Dil-oxLDL, L34358, Invitrogen) was adopted to trace the ox-LDL internalization. PMs from SFN- or vehicle-treated mice were incubated with Dil-oxLDL (10 µg/mL) for 6 h at 37 °C in a humidified 5% CO_2_ atmosphere (cell culture incubator). After washing 3 times with cold PBS, the uptake of ox-LDL in cells was visualized using a Leica confocal microscope. The mean fluorescent intensity was quantified using ImageJ.

### 2.12. Cholesterol Efflux Assay

PMs from SFN- or vehicle-treated mice were first labeled with 3-dodecanoyl-NBD cholesterol (1 μg/mL, 24618, Cayman Chemical, Ann Arbor, MI, USA) for 6 h. Next, the cells were washed with PBS and then incubated for 6 h in phenol red-free medium containing 0.2% BSA and apoA-I (10 µg/mL, A0722, Sigma) or HDL (50 µg/mL, L1567, Sigma). Negative control wells were only incubated with 0.2% BSA. The medium and cellular fluorescence-labeled cholesterol was analyzed using a fluorescence microplate reader (Bio-Tek instrument Inc., Winooski, VT, USA). The cholesterol efflux is expressed as the ratio of the medium fluorescence to the total fluorescence after subtracting the background of corresponding negative control.

### 2.13. Detection of Reactive Oxygen Species (ROS)

The intracellular ROS production was detected using an ROS-sensitive fluorescent dye, DCFH-DA. Briefly, THP-1 derived macrophages were seeded into 6-well plates (2 mL/well, 5 × 10^5^/mL) and mixed with DCFH-DA to a final concentration of 10 μM, incubated in the dark at 37 °C for 30 min and then washed with PBS 3 times. The cells were collected and centrifuged for 5 min at 100 *g*. The supernatant was then discarded, and the cells were resuspended in PBS in the dark. The fluorescence was analyzed by flow cytometry (Beckman Coulter, CA, USA).

### 2.14. Mitochondrial Membrane Potential (MMP) Assay

The MMP was assessed using the fluorescent probe JC-1. Differentiated THP-1 cells were seeded into 96-well plates (100 μL/well, 4.5 × 10^5^/mL) and exposed to various treatments. Subsequently, the medium was removed and replaced with 5 μg/mL JC-1. After 30 min of incubation at 37 °C in the dark, the fluorescence was determined using a fluorescence microplate reader (Bio-Tek instrument Inc, Winooski, VT, USA). The JC-1 fluorescence intensities of red aggregates and green fluorescence monomers were read at wavelengths of 530 nm and 590 nm, respectively. The MMP value was calculated as the ratio of red to green fluorescence.

### 2.15. Western Blotting

Aortas or macrophages were lysed with RIPA buffer (Beyotime, China) to extract the total protein. Equal amounts of protein (20 μg) were resolved in 8%~10% SDS–PAGE gels and then transferred onto polyvinylidene fluoride membranes (PVDF, Millipore, Billerica, MA, USA). After blocking with 5% nonfat milk for 2 h, the membranes were incubated with various specific primary antibodies: anti-CD36 (sc-7309; 1:1000; Santa Cruz Biotechnology), anti-PPARγ (sc-7273; 1:1000; Santa Cruz Biotechnology), anti-HO-1 (sc-390991; 1:1000; Santa Cruz Biotechnology), anti-SR-A (sc-166184; 1:1000; Santa Cruz Biotechnology), anti-β-actin (sc-8432; 1:1000; Santa Cruz Biotechnology), anti-Nrf2 (12721; 1:1000; Cell Signaling Technology, Danvers, MA, USA), anti-ABCA1 (ab18180; 1:500; Abcam) and anti-ABCG1 (ab52617; 1:1000; Abcam), followed by incubation with the corresponding horseradish peroxidase (HRP)-conjugated secondary antibodies. The protein bands were visualized using enhanced chemiluminescence (ECL) reagent (Thermo Fisher, San Jose, CA, USA), and densitometry was performed for quantification using ImageJ.

### 2.16. Statistical Analysis

The results are presented as the mean ± SEM. GraphPad Prism Version 8.0 was used for analysis. The statistical significance of differences between two groups was analyzed by unpaired two-tailed Student’s *t* tests, and one-way ANOVA with Bonferroni’s multiple-comparisons test was used for multiple-group comparisons. A value of *p* < 0.05 was considered to be significant.

## 3. Results

### 3.1. SFN Attenuates Atherosclerosis Progression and Improves the Serum Lipid Profile in ApoE^−/−^ Mice

**Figure 1:**
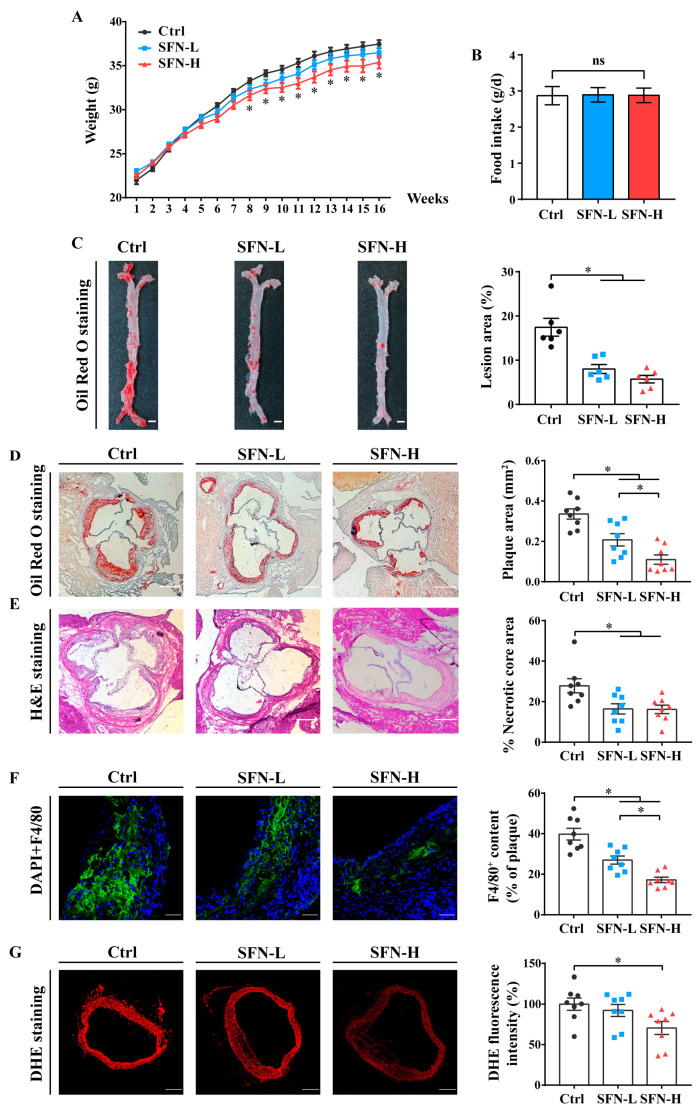
SFN attenuates atherosclerosis progression in ApoE^−/−^ mice. Eight-week-old male ApoE^−/−^ mice were fed a high-fat diet and subjected to oral gavage with 5 mg/kg SFN (SFN-L), 15 mg/kg SFN (SFN-H), or the vehicle (0.5% carboxymethylcellulose sodium (Ctrl)) every other day for 16 weeks (*n* = 12/group): (**A**) Bodyweights in the three groups were recorded every other day and compared every week; *n* = 12. (**B**) Twenty-four hour food intake of the ApoE^−/−^ mice; *n* = 12. (**C**) En face Oil Red O staining of the whole aorta. Lesion areas are expressed as the percentages of the en face aortic areas; scale bar = 2 mm; *n* = 6. (**D**) Oil Red O staining of representative aortic root sections. Lesion areas are expressed as mm^2^; scale bar = 200 μm; *n* = 8. (**E**) H&E staining of representative aortic root sections and quantification of the percentages of necrotic core areas within plaques; scale bar = 200 μm; *n* = 8. (**F**) Immunostaining of F4/80 and quantification of macrophage infiltration in aortic root lesions; scale bar = 50 μm; *n* = 8. (**G**) Representative fluorescence images and quantification of DHE-stained sections of aortic root; scale bar = 200 μm; *n* = 8. All the data are shown as the mean ± SEM. * *p* < 0.05: in panel A, a significant difference between SFN-H group and Ctrl group; in panel C-G, significantly different as indicated. ns: not significantly different.

To examine the preventive impact of SFN on atherosclerosis development, we fed ApoE^−/−^ mice a high-fat diet for 16 weeks, during which the vehicle alone (Ctrl), 5 mg/kg SFN (SFN-L), or 15 mg/kg SFN (SFN-H) was administered every other day. The bodyweights of the mice increased with age in all the groups and showed no significant differences between the Ctrl and SFN-L groups over the course of the experiment, but there was a slight reduction in the SFN-H group after 8 weeks. Meanwhile, no statistically significant difference in food intake was observed between the three groups (Figure 1A,B). Compared with the Ctrl group, the mice in the SFN-H group displayed lower serum levels of TC, TG and LDL-C but elevated levels of HDL-C, and those in the SFN-L group also had lower levels of LDL-C (Table 1).

SFN treatment reduced the atherosclerotic lesion area, as determined by the Oil Red O staining of the whole aorta and aortic root sections (Figure 1C,D). En face analysis of the entire aorta demonstrated that the atherosclerotic plaque size was markedly reduced by 53% and 66% in the SFN-L and SFN-H groups, respectively. Similar results were obtained from the quantitative analysis of the lesion area of the aortic sinus, which exhibited 38% and 65% reductions. Furthermore, the percentage of the necrotic core in the plaque area, a key index of plaque instability, was found to diminish to approximately 41% of the Ctrl for both doses of SFN, as judged by histological evaluation (Figure 1E). Additionally, the macrophage infiltration in the lesions, as identified by the immunohistochemical localization of the specific antigen F4/80 (a marker widely used to identify macrophages), was also prominently decreased in SFN-treated mice, and this effect appeared to be dose-dependent (Figure 1F). Moreover, we evaluated the vascular superoxide anion generation by the DHE staining of the cross-sections of the aortic sinus. Confocal microscopy analysis confirmed that the aortic oxidative stress was attenuated by SFN treatment, with a significant difference between the Ctrl and SFN-H groups (Figure 1G). Collectively, these findings suggest that SFN treatment can attenuate the progression of atherosclerosis.

### 3.2. SFN Inhibits ox-LDL-Induced Foam Cell Formation, ROS Generation and Mitochondrial Dysfunction in THP-1-Derived Macrophages

**Figure 2:**
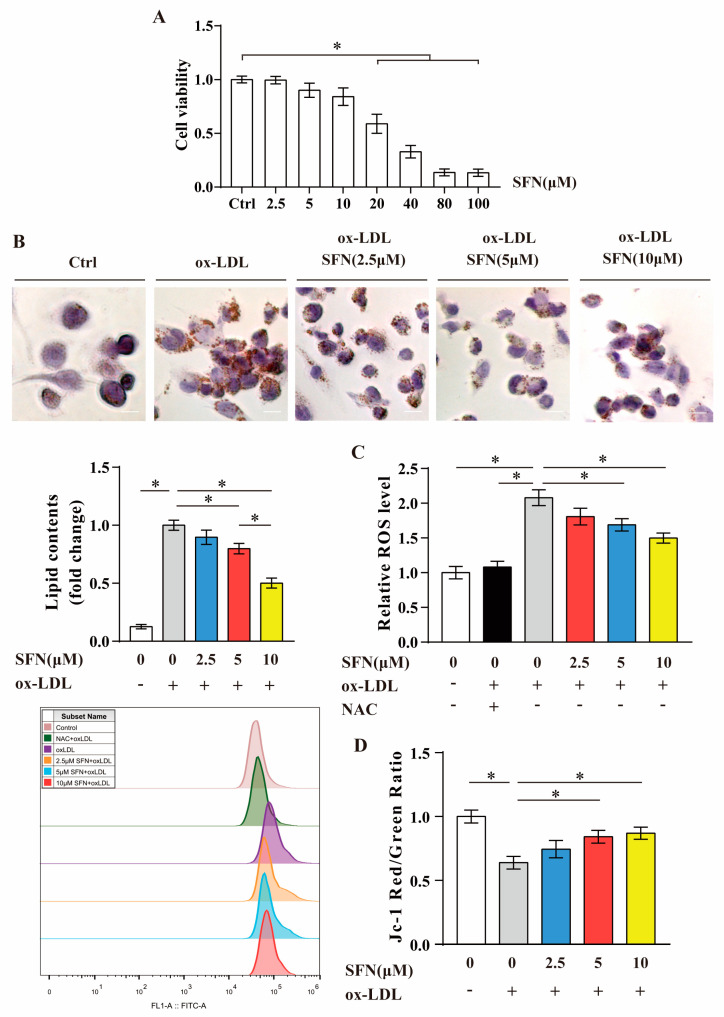
Inhibition of ox-LDL-induced foam cell formation, ROS generation and mitochondrial dysfunction by SFN pretreatment in THP-1-derived macrophages: (**A**) The cell viability of THP-1-derived macrophages exposed to serial concentrations of SFN (2.5~100 μM) or the vehicle for 24 h was measured by the WST-1 assay. (**B**–**D**) THP-1-derived macrophages were loaded with ox-LDL cholesterol (50 μg/mL) for 24 h after pretreatment with SFN (2.5, 5 and 10 μM) for 24 h. (**B**) Intracellular lipid accumulation was assessed by Oil Red O staining and quantification; scale bar = 10 μm. (**C**) Intracellular ROS levels were detected using a DCFH-DA probe and flow cytometry. A statistical graph and representative overlay histogram for the ROS generation are shown. (**D**) Quantitative analysis of mitochondrial membrane potential (MMP) was conducted using a JC-1 probe and fluorescence microplate reader, with the values calculated as a fluorescence intensity ratio of red to green. Data are expressed as the mean ± SEM of at least three independent experiments. * *p* < 0.05: significantly different as indicated.

The WST-1 assay results show that the biochemical half-maximal inhibitory concentration (IC_50_) value of SFN for differentiated THP-1 macrophages at 24 h was about 27.6 μM. Low doses (2.5~10 μM) of SFN showed no cytotoxicity (Figure 2A); thus, these were mainly used in the following experiments. The Oil Red O staining and quantification of the area of lipid droplets verified that loading THP-1-derived macrophages with ox-LDL (50 μg/mL, 24 h) significantly induced intracellular lipid deposition, which was significantly relieved by SFN pretreatment (20% at 5 μM and 49% at 10 μM) (Figure 2B). In addition, the influence of SFN on ROS production in ox-LDL-loaded macrophages was evaluated using a DCFH-DA fluorescent probe and flow cytometry analysis. As presented in Figure 2C, ox-LDL challenge dramatically elevated the intracellular ROS level, which was almost completely reversed by NAC pretreatment (used as a positive control) and significantly ameliorated by SFN pretreatment (19% at 5 μM and 28% at 10 μM). To evaluate the MMP, a voltage-sensitive JC-1 probe and fluorescence plate reader were used. The data showed that ox-LDL-loaded THP-1 macrophages exhibited a significant loss of MMP, and the abnormalities were significantly relieved under SFN pretreatment (31% at 5 μM and 36% at 10 μM) (Figure 2D). Taken together, the in vitro findings suggest that SFN may protect against ox-LDL-evoked lipid accumulation in macrophages and the consequent cell injury.

### 3.3. SFN Inhibits Macrophages’ Cholesterol Uptake and CD36 Expression in ApoE^−/−^ Mice

**Figure 3:**
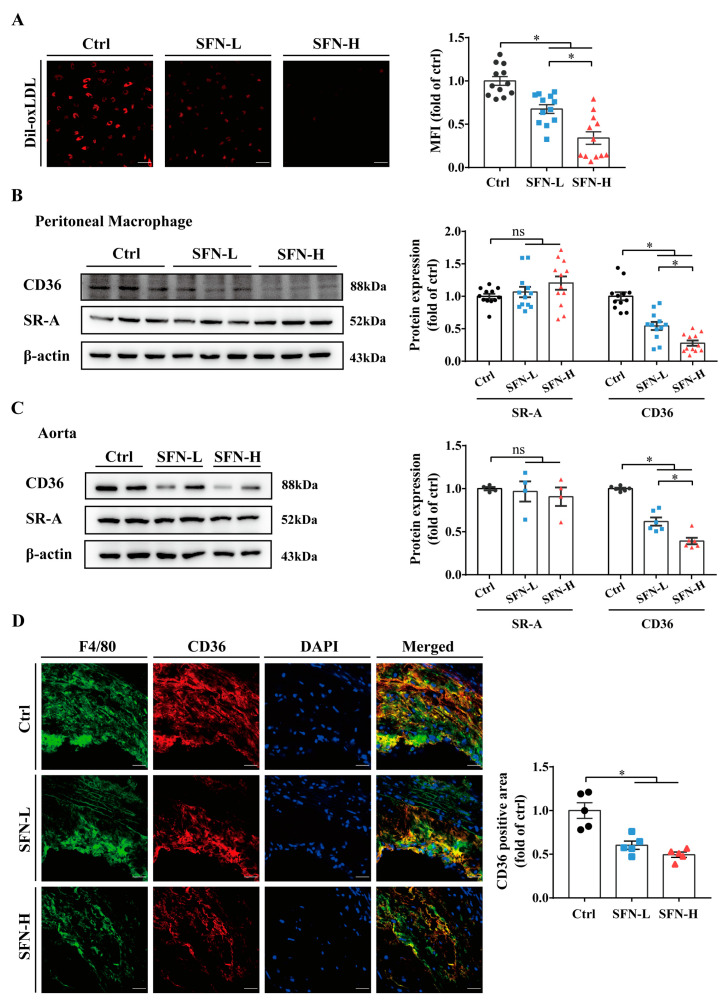
SFN inhibits macrophages’ cholesterol uptake and CD36 expression in ApoE^−/−^ mice. Eight-week-old male ApoE^−/−^ mice were fed a high-fat diet and subjected to oral gavage with 5 mg/kg SFN (SFN-L), 15 mg/kg SFN (SFN-H), or the vehicle (0.5% carboxymethylcellulose sodium (Ctrl)) every other day for 16 weeks (*n* = 12/group). Three days before euthanasia, the mice were intraperitoneally injected with thioglycolate, and peritoneal macrophages (PMs) were obtained by peritoneal lavage at the end of the experiment: (**A**) The cholesterol uptake of the isolated PMs was determined using Dil-oxLDL, followed by quantitative analysis of the intracellular MFI; scale bar = 50 μm; *n* = 12. (**B**,**C**) Western blot detection and band densitometric analysis of CD36 and SR-A protein expression in (**B**) PMs (*n* = 12) and in (**C**) aortas (*n* = 6). (**D**) CD36 expression was detected by immunofluorescence staining and colocalized with F4/80+ macrophages in atherosclerotic lesions; scale bar = 50 μm. The reported values are the fold changes in the CD36 and F4/80 double-positive areas relative to the control; *n* = 5. All the data are shown as the mean ± SEM. * *p* < 0.05: significantly different as indicated; ns: not significantly different; MFI: mean fluorescence intensity.

To further clarify the mechanism underlying the preventive effects of SFN on macrophage foam cell formation and atherosclerosis progression, we collected PMs from ApoE^−/−^ mice at the end of experiment and assessed their ox-LDL phagocytotic activities using Dil-labeled ox-LDL. The ex vivo studies revealed that the cholesterol uptakes by PMs in the SFN-L and SFN-H groups were decreased by 32% and 66%, respectively (Figure 3A). Considering that SR-A and CD36 play major roles in mediating ox-LDL uptake, we further explored their expression in PMs. Western blot analysis indicated that the administration of SFN to ApoE^−/−^ mice for 16 weeks dose-dependently reduced the expression of CD36 in resident PMs but exerted no significant impact on the expression of SR-A (Figure 3B). Similar results were found from Western blots analyzing expression in the whole aorta (Figure 3C). To further determine the impact of SFN on macrophages’ CD36 expression in situ, we performed dual immunofluorescent staining with anti-CD36 and F4/80 antibodies. Colocalization analysis revealed a statistically significant decrease in CD36 expression in intraplaque macrophages in both the SFN-L and SFN-H groups compared with the Ctrl group (Figure 3D). Altogether, these data demonstrate that SFN can suppress the engulfment of ox-LDL by macrophages through mechanisms that might involve the downregulation of CD36 expression.

### 3.4. SFN Promotes Macrophages’ Cholesterol Efflux and ABCA1/G1 Expression in ApoE^−/−^ Mice

**Figure 4:**
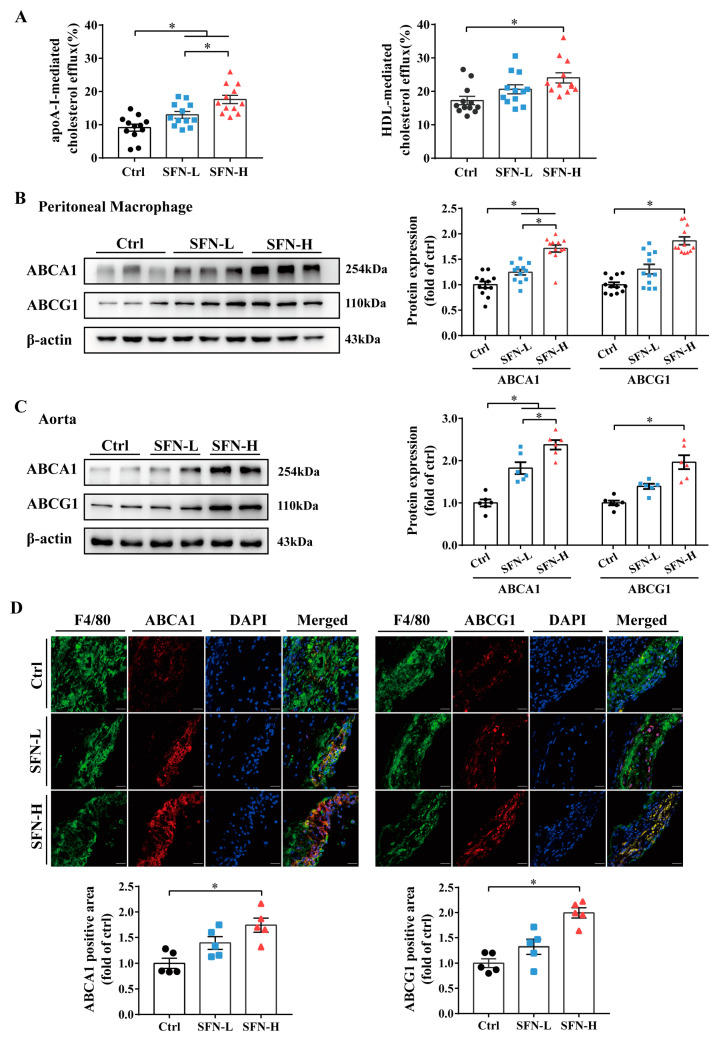
SFN promotes macrophages’ cholesterol efflux and ABCA1/G1 expression in ApoE^−/−^ mice. ApoE^−/−^ mice were treated, and PMs were isolated, as shown in Figure 3, before the following analysis: (**A**) Upon equilibration with NBD-cholesterol for 6 h and incubation with apoA-I or HDL for another 6 h, the cholesterol efflux from PMs was measured using a fluorometer and is expressed as the percentage of fluorescence in the medium relative to the total amount of fluorescence; *n* = 12. (**B**,**C**) Western blot detection of protein abundance of ABCA1 and ABCG1 in (**B**) PMs (*n* = 12) and in (**C**) aortas (*n* = 6). (**D**) Cross-sections of aortic sinuses stained for ABCA1, ABCG1 and F4/80 and quantitative analysis of F4/80+ ABCA1+ or F4/80+ ABCG1+ area; scale bar = 50 μm; *n* = 5. The results are presented as the mean ± SEM. * *p* < 0.05: significantly different as indicated.

Since the formation of macrophage foam cells is influenced by the balance between modified LDL uptake and cholesterol efflux, we investigated the effects of SFN on cholesterol efflux and the expression of ABCA1 and ABCG1 in this atherosclerosis-prone genetic mouse model. PMs from SFN- or vehicle-treated ApoE^−/−^ mice were incubated with NBD-cholesterol for 6 h, and the apoAI- and HDL-mediated NBD-cholesterol effluxes were measured. We found that SFN dose-dependently increased apoAI-mediated cholesterol efflux, and at high doses (15 mg/kg), it also significantly increased HDL-mediated efflux (Figure 4A). Comparable increases in the expression of ABCA1 and ABCG1 protein were simultaneously observed in PMs from SFN-treated mice compared to the vehicle control mice (Figure 4B), which was further confirmed by Western blot analysis for aortas (Figure 4C). Likewise, dual immunofluorescent assays showed that the mice in the SFN-H group had increased expression of both ABCA1 and ABCG1 in F4/80+ cells within plaques (Figure 4D). These results suggest that the targeted promotion of ABCA1/G1-mediated macrophages’ cholesterol efflux may constitute another mechanism by which SFN protects against foam cell formation and atherosclerosis.

### 3.5. Downregulation of PPARγ and Upregulation of Nrf2/HO-1 Expression by SFN in ApoE^−/−^ Mice

**Figure 5:**
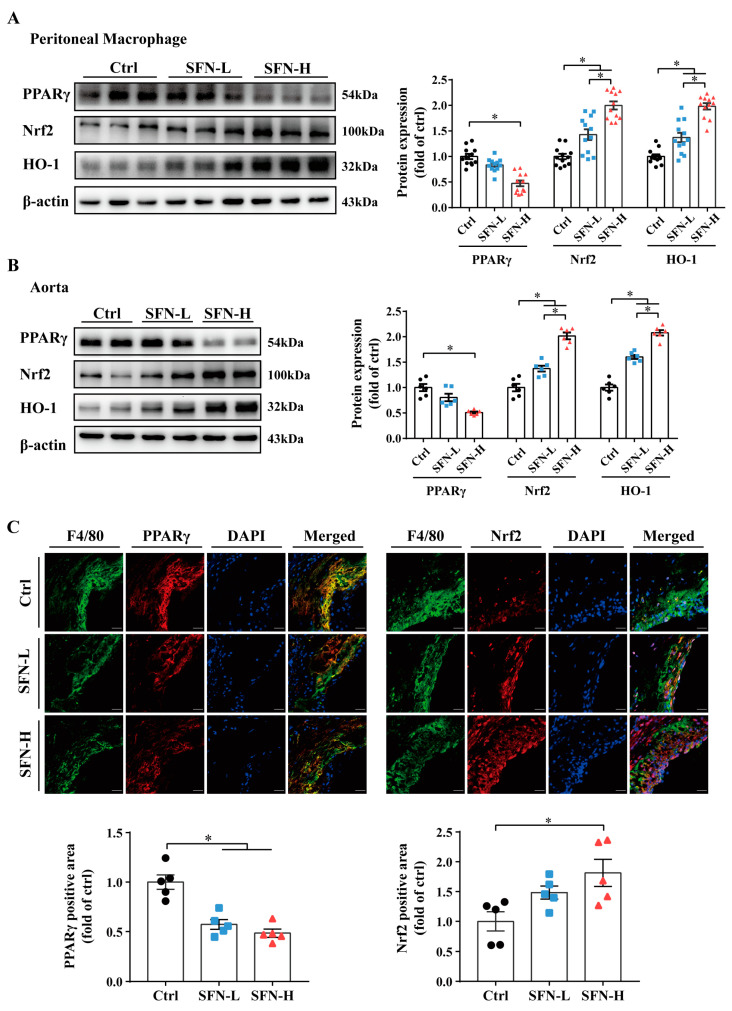
SFN downregulates PPARγ expression and upregulates Nrf2/HO-1 expression in ApoE^−/−^ mice. ApoE^−/−^ mice were treated, and PMs were isolated, as shown in Figure 3, before the following analysis: (**A**,**B**) Immunoblots showing levels of PPARγ, Nrf2 and HO-1 in (**A**) PMs (*n* = 12) and in (**B**) aortas (*n* = 6). (**C**) Immunofluorescence analysis of Nrf2 and PPARγ protein expression in F4/80+ cells in aortic sinus sections. The nuclei were stained with DAPI; scale bar = 50 μm; *n* = 5. Data are presented as the mean ± SEM. * *p* < 0.05: significantly different as indicated.

Both PPARγ and Nrf2 have been reported to participate in the transcriptional regulation of CD36 and ABCA1/G1. Additionally, Nrf2 and its representative downstream target heme oxygenase-1 (HO-1) have been implicated in macrophages’ phenotypes and atherosclerosis. Thus, their expression in PMs and aortic tissues following SFN treatment was measured. Interestingly, the results reveal an opposite action of SFN on PPARγ and Nrf2/HO-1. Significant dose-dependent upregulation of Nrf2/HO-1 protein expression was observed in PMs and whole aorta tissues from SFN-treated ApoE^−/−^ mice. Conversely, PPARγ expression was found to be downregulated by SFN in PMs and whole aorta, with a statistically significant difference between the SFN-H and Ctrl groups (Figure 5A,B). Furthermore, coimmunofluorescent staining with F4/80 corroborated SFN’s stimulatory effect on Nrf2 and inhibitory effect on PPARγ in intraplaque macrophages (Figure 5C). These results provide direct evidence that the levels of Nrf2 and PPARγ in macrophages were oppositely modulated, underlying SFN’s atheroprotective effect.

## 4. Discussion

Many studies have indicated that ITCs have protective effects regarding cardiovascular metabolic diseases. However, investigations targeting macrophage foam cell formation to specifically evaluate the atheroprotective potency of ITCs are still lacking. In the present study, high fat diet fed ApoE^−/−^ mice and ox-LDL-overladen THP-1-derived macrophages were subjected to SFN treatment. We demonstrate that SFN can play a preventive role in macrophage foam cell formation and atherosclerosis through restraining cholesterol uptake and inducing efflux via the potential modulation of the expression of CD36, ABCA1/G1, PPARγ and Nrf2/HO-1.

We have demonstrated that orally administering SFN to high fat diet fed ApoE^−/−^ mice notably inhibited atherosclerotic plaque formation and ameliorated the unstable phenotype, as manifested by reductions in the atherosclerotic lesion area, intralesional macrophage infiltration and necrotic core sizes, as well as lower levels of ROS in aortic walls (Figure 1). Meanwhile, the dyslipidemia was remarkably improved (Table 1). Beneficial effects of SFN on lipid profiles have been well established in many metabolic disease models and meta-analyses [65]. However, no research has reported the action of SFN on atherosclerotic plaque formation to date. Nevertheless, several investigations have proved that SFN interventions protect against neointima formation/neointimal hyperplasia, reduce the intima/media (I/M) ratio and inhibit vascular injury by injecting inflammatory factors [30,31], applying surgical interventions and using high-fat/cholesterol-fed murine models as well as hypercholesterolemic rabbits [29,32,33,34,35]; however, no study provides mechanistic perspectives regarding foam cells. Notably, two relevant studies provide direct evidence that allyl isothiocyanate (AITC) and JC-5411, a phenethyl isothiocyanate (PEITC) formulation, inhibit atherosclerotic plaque formation in ApoE^−/−^ mice [66,67]. However, the former study mainly focused on transient receptor potential ankyrin 1 channel (TRPA) I, and the latter paid close attention to lipid metabolism and antioxidant effects in the liver and inflammation in endothelial cells (ECs), but it did not explore the direct influence of these ITCs on macrophage foam cell formation and their involvement in lesions. Intriguingly, SFN-induced reductions in macrophage infiltration in vessel walls have been also observed in TNF-α-treated mice and spontaneously hypertensive stroke-prone (SHRSP) rats [31,68]. Our study, using well-defined classical ApoE^−/−^ mice, provides the first direct evidence that SFN exerts an antiatherosclerotic effect.

Macrophage foaming, as the central event in atherosclerosis, is a very important and attractive field of research. Indeed, it has been confirmed to be targeted by many bioactive food ingredients and medicines [8,69,70], but whether SFN can affect it remains unknown. In order to better assess the possible atheroprotective mechanism of SFN involving foam cell formation, we carried out an in vitro study using ox-LDL-laden THP-1-derived macrophages and revealed that the pre-administration of SFN at noncytotoxic doses had an inhibitory effect. In addition to inducing intracellular lipid accumulation, loading with ox-LDL also resulted in oxidative stress and collapse of the MMP in THP-1-derived macrophages; both effects were inhibited by SFN pretreatment (Figure 2). Lipid deposition, ROS generation and mitochondrial dysfunction in macrophages can lead to multiple downstream events, such as the activation of inflammasomes and increases in the expression and release of inflammatory cytokines and proteolytic enzymes, promoting ox-LDL uptake, impairing cholesterol efflux and inducing macrophage trapping and apoptosis, which, together, facilitate the expansion of atherosclerotic lesions and increase their instability [5,6,7,9]. Specifically, an elaborate study revealed that ox-LDL/CD36 signals in macrophages induce abnormal fatty acid metabolism that is correlated with mitochondria-derived oxidative stress, thus eliciting chronic inflammation [71]. Our in vitro study confirms that SFN pretreatment can inhibit ox-LDL-induced foam cell formation and lipo-toxicity, which may constitute the main mechanisms by which SFN protects against atherosclerosis.

Cholesterol influx and efflux are key determinants of foam cell formation and atherogenesis. Transmembrane cholesterol transportation involves multiple mechanisms, with SR-A, CD36 and ABCA1/G1 regarded as principal contributors. Herein, by using a high fat diet fed ApoE^−/−^ mouse model, we demonstrated that oral SFN treatment significantly suppressed cholesterol engulfment by PMs while promoting its outflow from PMs to both apoA-I and HDL, accompanied by reduced CD36 but unchanged SR-A protein expression and increased ABCA1/G1. Moreover, SFN was found to similarly modulate the protein expression of the above-mentioned cholesterol-trafficking proteins within intraplaque macrophages and in whole aortic tissues (Figure 3 and Figure 4). Notably, a recent study has reported that PEITC protects against lipid deposits in THP-1-derived macrophages co-exposed to ox-LDL and lipopolysaccharide (LPS); the downregulation of CD36, SR-A1 and lectin-like oxidized low-density lipoprotein receptor-1 (LOX-1) and upregulation of ABCA1 protein expression were simultaneously observed [72]. Additionally, in high-fat/cholesterol-diet C57BL/6 mice, the research team observed that PEITC was just as effective in modulating CD36, SR-A1 and ABCA1 protein expression and lipid accumulation in the liver [73]. Augmented ABCA1 protein levels in the liver have also been observed in JC-5411-treated ApoE^−/−^ mice [67]. Additionally, an AITC-induced reversal of ABCA1/G1 downregulation was observed in the rat alveolar macrophage cell line NR8383 challenged to a cigarette smoke extract (CSE) and in the lung tissues of chronic obstructive pulmonary disease (COPO) rats [74]. Interestingly, reduced lipid deposition and CD36 levels in the kidneys were detected in an SFN-treated unilateral ureteral obstruction-induced renal damage rat model [75]. Nevertheless, the limited reports on the impact of SFN on CD36 are inconsistent [76,77,78,79,80]. Accumulating evidence has shown that SFN could improve the capacity of macrophages to phagocytose, clear, or kill various pathogens. Additionally, the underlying mechanism may involve the regulation of CD36, Nrf2 and macrophage receptor with collagenous structure (MARCO). In light of the diversity and complexity of SR ligands, the ability of SFN to modulate CD36 and other SRs, as well as the consequences of this, deserve further investigation [81,82,83,84]. In short, our in vivo findings suggest that SFN treatment can concomitantly inhibit cholesterol uptake by and promote cholesterol export from macrophages by mechanisms involving the downregulation of CD36 expression and upregulation of ABCA1/G1 expression.

Both Nrf2 and PPARγ are important sensors of lipid disturbances and stress responses, and they have been involved in macrophage lipid accumulation and atherosclerosis [6,38,43,44,85]. Unsurprisingly, as a classic Nrf2 activator, SFN significantly induced Nrf2 activation in macrophages, regardless of whether they were from the peritoneal cavity or were trapped in plaques, as shown by the elevated expression of Nrf2 protein and its downstream target HO-1. These findings were also corroborated by protein analysis in the aorta (Figure 5). The age-related impairment of Nrf2 signaling is related to aging and atherosclerosis [86,87]. Moreover, it is reported that an atherosclerosis-susceptible site of the murine aorta displays lower Nrf2 expression in ECs and more pronounced activation upon SFN treatment compared with the protected region [88]. Our findings suggest that the activation of Nrf2 in macrophages represents one of the most important specific antiatherosclerotic effects of SFN. Interestingly, although positive feedback regulation and a cooperative effect of PPARγ and Nrf2 have been proposed [57,60], we found opposite changes in their protein expression in ApoE^−/−^ mice following SFN treatment at the three levels (Figure 5). PPARγ is mainly expressed in adipocytes, monocytes/macrophages, the liver, skeletal muscle and the heart, and it has been suggested to mediate adipogenesis and be related to ectopic lipid accumulation, such as that occurring in liver steatosis, cardiac lipotoxicity and atherosclerosis [55,56]. The inhibitory effect of SFN on macrophages’ PPARγ expression observed here is in line with several discoveries in hepatocytes and adipocytes [61,62,63,64], and it is supported by a study using THP-1-derived macrophages [89], but it is contrary to the results of the preventive effect of PEITC on macrophage foam cell formation [72]. There is evidence that PPARγ may induce ABCA1/G1 and cholesterol removal from macrophages through a liver X receptor (LXR)-mediated transcriptional cascade [8], but a study revealed that PPARγ activation did not contribute to the expression of macrophage ABCA1 or the cholesterol efflux that was mediated by ABCA1 [90]. Notably, the modulation of PPARγ–CD36 pathways by various pharmaceutical agents, natural products and their extracts has been widely reported to blunt foam cell formation [69,70,91,92,93,94]. Our findings, along with the others, support the inference that the inhibition of PPARγ by SFN in macrophages may play a remarkable role in its modulation of CD36 and cholesterol influx, independently of its effects on ABC transporters and cholesterol outflow.

Like PPARγ, Nrf2 has been proposed to exert a dual influence on foam cells, since it has been shown to both promote cholesterol intake via transactivating CD36 and induce cholesterol efflux via increasing ABCA1/G1 expression [38,43]. However, the data on Nrf2’s role in the pathogenesis of atherosclerosis and modulation of CD36 expression and foam cell formation remain controversial, and our data presented here clearly demonstrate that SFN, a potent Nrf2 activator, does not promote macrophages’ expression of CD36 and internalization of cholesterol. The activation of Nrf2/HO-1 has been suggested to be responsible for the increase in ABCA1/G1 expression induced by tert-butylhydroquinone (tBHQ) and trans-3,5,4′-trimethoxystilbene (TMS), a novel derivative of resveratrol, respectively [46,47], and mediate the antiatherosclerotic efficacy of a chalcone derivative, 1m-6, by mechanisms involving the stabilization of ABCA1 mRNA and potential ABCA1-regulating miRNAs [48]. The activation of Nrf2 and subsequent inhibition of NF-κB signaling by epigallocatechin-3-gallate (EGCG) were found to alleviate the TNF-α-caused inhibition of ABCA1 gene expression and cholesterol efflux [49]. The synergistic effects of Nrf2 and sirtuin 1 (Sirt1) in inducing peroxiredoxin 1 (Prdx1)/ABCA1 expression were reported to underlie tanshindiol C’s (Tan C) ability to protect against macrophage foam cell formation [50]. Interestingly, other members of the ABC transporter family such as multidrug resistance protein (MRP) and ABCB10 have been proven to be regulated by Nrf2 [95,96]. Therefore, we speculate that Nrf2 may act as a principal mediator of the SFN-induced increase in ABCA1/G1 expression and cholesterol efflux.

It has been suggested that hundreds of genes can be activated by SFN via Nrf2, among which HO-1 is a major representative. HO-1 has antioxidant, anti-inflammatory, antiapoptotic and antithrombotic activity, hence helping to protect against atherosclerosis and gaining attention as a potential therapeutic target with clinical significance [85]. Moreover, among the several identified macrophage phenotypes, M(Hb), Mhem and Mox highly express HO-1. The first two are considered antiatherogenic subsets, but the last is less well-defined [38,97,98]. Hence, we believe that the protective effect exerted by SFN on macrophage foam cell formation and atherosclerosis was, at least in part, mediated by the induction of HO-1 expression.

Although our study has some innovative findings, it still has limitations, including the following: (1) Only male ApoE^−/−^ mice were selected in the experiment; however, sex is a biological variable of atherosclerosis. Hence, rigorously designed and conducted sex difference research is needed. (2) We just focused on the effect of SFN on macrophage foam cell formation and atherosclerosis from the perspective of cholesterol uptake and efflux, but the intracellular cholesterol events were not uncovered, such as cholesterol esterification and hydrolysis, cholesterol synthesis and translocation. (3) Mechanistic implications of other cholesterol transporters and related mediators, as well as nonmacrophages, cannot be excluded.

## 5. Conclusions

In conclusion, our investigation confirms that in male ApoE^−/−^ mice, chronic and low-dose oral SFN administration may potently inhibit atherosclerotic plaque formation and instability by mechanisms involving the regulation of macrophages’ cholesterol-handling capacity and cholesterol accumulation. SFN supplementation may induce a phenotypic shift of both intralesional and intraperitoneal macrophages toward high expressions of Nrf2/HO-1 and ABCA1/G1 and low expressions of PPARγ and CD36. Consistently, functional studies of PMs reveal that SFN can regulate cholesterol flux in a dual manner, namely repressing influx and promoting efflux. These findings extend the current mechanistic insights into SFN’s ability to help prevent cardiometabolic diseases and support the health benefits of high intakes of cruciferous vegetables.

## Figures and Tables

**Table 1:** Serum lipid profile in high fat diet fed ApoE^−/−^ mice.

Groups	TC	TG	LDL-C	HDL-C
	(mmol/L)	(mmol/L)	(mmol/L)	(mmol/L)
Ctrl	17.04 ± 2.52	2.21 ± 0.37	14.52 ± 2.60	1.47 ± 0.27
SFN-L	14.58 ± 2.06	1.82 ± 0.27	11.23 ± 1.27 ^1^	1.81 ± 0.24
SFN-H	11.67 ± 1.82 ^1^	1.39 ± 0.18 ^1^	9.61 ± 1.09 ^1^	2.19 ± 0.21 ^1^

Data are expressed as the mean ± SEM; *n* = 12. TC: total cholesterol; TG: triglyceride; LDL-C: low-density lipoprotein cholesterol; HDL-C: high-density lipoprotein cholesterol. ^1^ *p* < 0.05 vs. Ctrl group.

## Data Availability

The data are contained within the article.
